# Pathogen-specific mortality in very low birth weight infants with primary bloodstream infection

**DOI:** 10.1371/journal.pone.0180134

**Published:** 2017-06-22

**Authors:** Brar C. Piening, Christine Geffers, Petra Gastmeier, Frank Schwab

**Affiliations:** Institute for Hygiene and Environmental Medicine, Charité University Medical Center, Berlin, Germany; Centre Hospitalier Universitaire Vaudois, FRANCE

## Abstract

**Objective:**

Mortality in very low birth weight infants following microbiology confirmed primary bloodstream infections varies with the type of causative pathogen. Given evidence from other studies that infections with gram negative bacteria and fungi cause a higher case fatality risk. We tried to confirm this in a nation-wide multi-center trial.

**Methods:**

A cohort of 55,465 very low birth weight infants from 242 neonatal departments participating in the German national neonatal infection surveillance system NEO-KISS was used to investigate differences in the case fatality risk of microbiology confirmed primary bloodstream infections according to individual pathogens. Cox proportional hazard regression analyses were performed with the outcomes death and time from microbiology confirmed primary bloodstream infections. The results were adjusted to the recorded risk factors and hospital and department characteristics.

**Results:**

A total of 4 094 very low birth weight infants with microbiology confirmed primary bloodstream infections were included in the analysis. The crude case fatality risk was 5.7%. The Cox proportional hazard regression analysis with adjustment for available risk factors revealed that microbiology confirmed primary bloodstream infections caused by *Klebsiella spp*. (HR 3.17 CI95 1.69–5.95), *Enterobacter spp*. (HR 3.42 CI95 1.86–6.27), *Escherichia coli* (HR 3.32 CI95 1.84–6.00) and *Serratia spp*. (HR 3.30 CI95 1.44–7.57) were associated with significantly higher case fatality risk compared to *Staphylococcus aureus*. After adjusting, case fatality risk of *Candida albicans* causing microbiology confirmed primary bloodstream infections was not higher than that of *S*. *aureus*.

**Conclusion:**

In very low birth weight infants, bloodstream infections caused by gram negative pathogens have an increased case fatality risk compared to bloodstream infections caused by gram positive pathogens. This should be considered for prevention and therapy. Further research should address the specific risk factors for case fatality of *C*. *albicans* bloodstream infections.

## Introduction

Nosocomial bloodstream infections in infants with a birthweight below 1500 g (very low birth weight infants; VLBW) are an important cause of neonatal mortality. Case fatality risk (CFR) of these infections depends on patient factors, department factors and the type of pathogen. Earlier studies identified a higher CFR of nosocomial bloodstream infections when the causing pathogens were gram negative bacteria or fungi [1], [2], [3]. Three studies even determined a significant difference between gram positive and gram negative pathogens after adjusting for several risk factors [4], [5].[6] Multidrug resistant pathogens have also been shown to be associated with increased mortality [6–8]. However, only two published studies investigated the differences of various pathogen species in terms of mortality [6, 9] which is probably due to the large sample sized needed to perform such an analysis.

## Patients and methods

The surveillance component for VLBW infants of the German national nosocomial infection surveillance system (NEO-KISS) is one of the largest databases for health care associated infections in neonates worldwide [10, 11]. A total of 242 neonatal departments are participating and providing their data on a regular basis for ongoing quality assurance. Therefore, this database offers a good opportunity to determine the influence of specific pathogens on the CFR of nosocomial bloodstream infections in VLBW infants. All data in the NEO-KISS database were obtained from medical records in a fully anonymized and de-identified manner according to German data protection laws. None of the researchers and authors had access to identifying information.

The methods and definitions of NEO-KISS are described elsewhere [10]. According to the surveillance protocol, laboratory confirmed and clinical cases of late-onset (>72h postnatal) primary bloodstream infections are recorded and the follow up of patients ends with the occurrence of first of the following three events: 1. the actual weight of the child reaches or exceeds 1800g; 2. the child is transferred to another department; 3. the child dies.

This analysis focuses on microbiology confirmed primary bloodstream infections (MCBSI) only. Their definitions are as follows:

1. A recognized pathogen other than *coagulase-negative staphylococci* (*CoNS*) cultured from blood or cerebrospinal fluid (CSF; this is included in the definition because in this age group meningitis usually develops haematogenously); and at least two of the following signs: temperature >38 or <36.5°C or temperature instability, tachycardia or bradycardia, apnoea, extended capillary refill time (CRF), metabolic acidosis, hyperglycaemia, other sign of sepsis such as apathy; and the organism is not related to an infection at another site.
2. *Coagulase negative Staphylococcus (*CoNS) is cultured from blood; and at least two of the following signs: temperature >38 or <36.5°C or temperature instability, tachycardia or bradycardia, apnoea, extended CRF, metabolic acidosis, hyperglycaemia, other sign of sepsis such as apathy; and patient has one of the following signs: C-reactive protein >2.0 mg/dL, immature/total neutrophil ratio (I/T ratio) >0.2, leukocytes <5/nL, platelets <100/nL;and the organism/infection is not related to an infection at another site

VLBW infants with MCBSI recorded in NEO-KISS in the period from January 1^st^ of 2005 to December 31^st^ of 2013 were used for this study. The following exclusion criteria were used:

- Patients with length of stay of less than 3 days (MCBSI happening within the first 3 days after admission is excluded per protocol so we also had the exclude those patients from the denominator who couldn’t acquire a MCBSI by definition)
- Patients with MCBSI with incorrect/missing date of infection
- Patients with MCBSI with more than one microorganism specified
- Patients with missing data about the relevant risk factors gestational age, mode of delivery and gender

**Fig 1:**
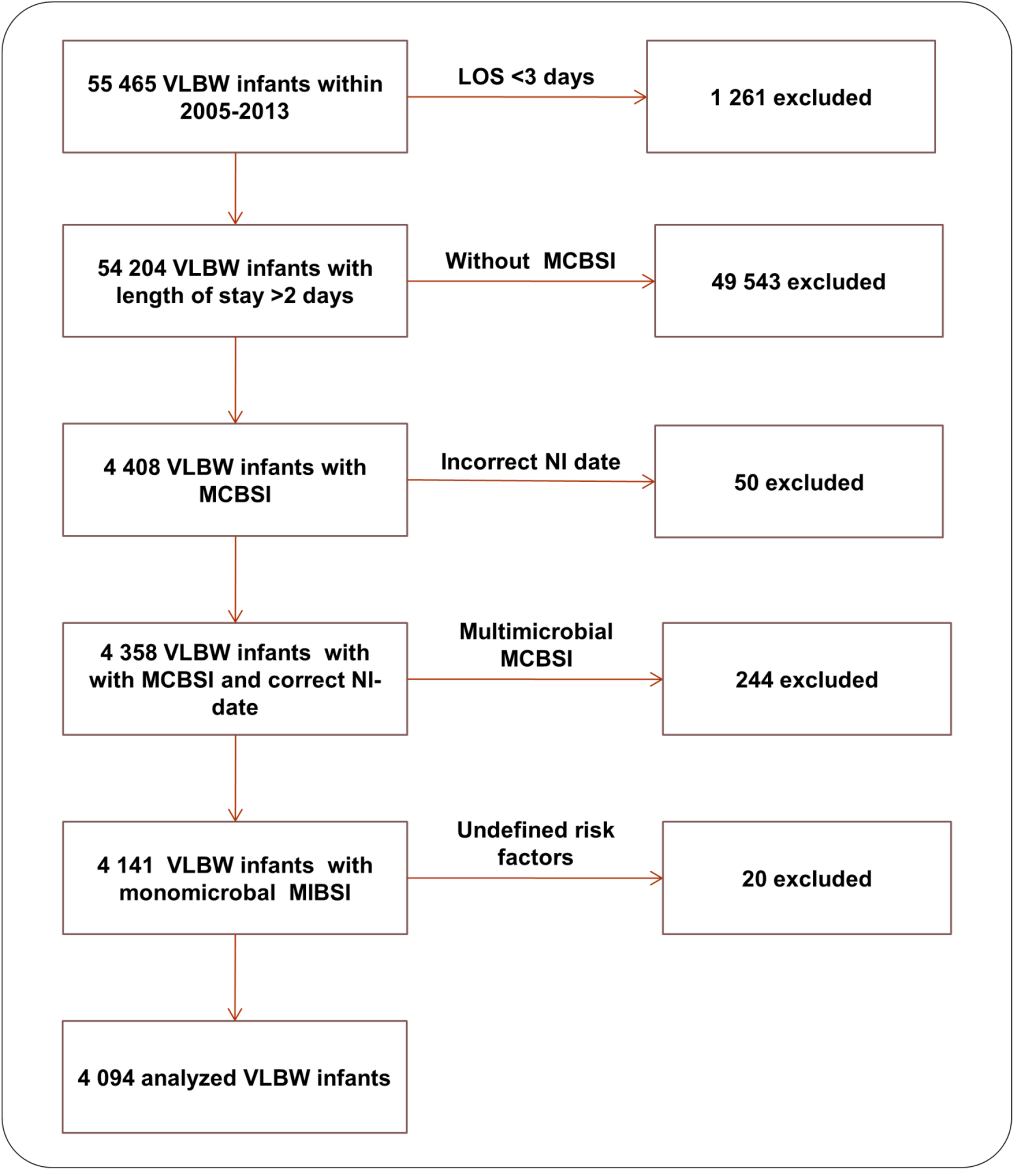
Exclusion flow chart. LOS, length of stay; VLBW, very low birth weight; NI, nosocomial infection; MCBSI, microbiological confirmed bloodstream infection.

A flow diagram with included VLBW infants with MCBSI in the analysis is shown in Fig 1.

In the descriptive analysis (Table 1), numbers and percent values were calculated; differences were tested by Chi-square test. In the multivariable analysis, Cox proportional hazard regression analysis was performed with the outcome death and the time (days) from the date of the last MCBSI until the end of follow up which may happen due to one of the following three reasons according to the NEO-KISS protocol: 1. death, 2. achievement of a weight of 1800g or 3. discharge or transfer from the department. Cox regression (or proportional hazards regression) is a method for investigating the effect of several variables on the time a specified event (e.g. mortality) takes to happen. We calculated hazard ratios with 95% confidence intervals where the hazard ratio can be interpreted as the predicted change in the hazard for a category to the reference category in the analyzed variable.

**Table 1:** Characteristics of neonates who had at least one microbiology confirmed bloodstream infection (MCBSI) after they had been admitted to the NEO-KISS department and before they had been discharged or their actual weight had exceeded 1800 g (whichever happened first).

Parameter	Category	Total number	%	Total number died	%	p-value	Crude CFR (%)
patients	total	4094	100.0%	234	100.0%		5.7%
type of pathogen	*CoNS*	2301	56.2%	58	24.8%	<0.001	2.5%
	*S*. *aureus*	392	9.6%	16	6.8%		4.1%
	*E*. *coli*	226	5.5%	39	16.7%		17.3%
	*Enterobacter spp*.	207	5.1%	35	15.0%		16.9%
	*Enterococcus spp*.	191	4.7%	12	5.1%		6.3%
	*Klebsiella spp*.	170	4.2%	27	11.5%		15.9%
	*C*. *albicans*	91	2.2%	15	6.4%		16.5%
	*Serratia spp*.	50	1.2%	9	3.8%		18.0%
	other pathogens	466	11.4%	23	9.8%		4.9%
gender	male	2216	54.1%	128	54.7%	0.856	5.8%
	female	1878	45.9%	106	45.3%		5.6%
gestational age (weeks)	<24	250	6.1%	45	19.2%	<0.001	18.0%
	24–25	1119	27.3%	116	49.6%		10.4%
	26–27	1022	25.0%	29	12.4%		2.8%
	28–29	932	22.8%	23	9.8%		2.5%
	>29	771	18.8%	21	9.0%		2.7%
birth weight (grams)	<500	269	6.6%	43	18.4%	<0.001	16.0%
	500–749	1208	29.5%	118	50.4%		9.8%
	750–999	1136	27.7%	38	16.2%		3.3%
	1000–1249	782	19.1%	19	8.1%		2.4%
	1250–1499	699	17.1%	16	6.8%		2.3%
mode of delivery	Caesarean Section	3297	80.5%	155	66.2%	<0.001	4.7%
	Vaginal	523	12.8%	55	23.5%		10.5%
	Emergency Caesarean Section	274	6.7%	24	10.3%		8.8%
multiple birth	n. d.	38	0.9%	3	1.3%	0.844	7.9%
	single	2882	70.4%	164	70.1%		5.7%
	multiple	1174	28.7%	67	28.6%		5.7%
appropriateness for gestational age	AGA	2923	71.4%	161	68.8%	<0.001	5.5%
	SGA	1008	24.6%	58	24.8%		5.8%
	LGA	139	3.4%	9	3.8%		6.5%
	GW< = 22 or n. d	24	0.6%	6	2.6%		25.0%
Device association	CVC	2029	49.6%	165	70.5%	<0.001	8.1%
	PVC	1589	38.8%	52	22.2%		3.3%
	no device	476	11.6%	17	7.3%		3.6%
time from admission to BSI	<8 days	350	8.5%	51	21.8%	<0.001	14.6%
	8–14 days	1576	38.5%	91	38.9%		5.8%
	15–21 days	900	22.0%	35	15.0%		3.9%
	> = 22 days	1268	31.0%	57	24.4%		4.5%
Birth Location	n. d.	258	6.3%	19	8.1%	0.044	7.4%
	Inhouse Birth	3605	88.1%	195	83.3%		5.4%
	Immediate Postnatal Transport	130	3.2%	14	6.0%		10.8%
	Longterm Postnatal Transport	101	2.5%	6	2.6%		5.9%
year of birth	2005	188	4.6%	13	5.6%	0.054	6.9%
	2006	363	8.9%	20	8.5%		5.5%
	2007	552	13.5%	42	17.9%		7.6%
	2008	489	11.9%	39	16.7%		8.0%
	2009	545	13.3%	29	12.4%		5.3%
	2010	528	12.9%	31	13.2%		5.9%
	2011	488	11.9%	22	9.4%		4.5%
	2012	464	11.3%	19	8.1%		4.1%
	2013	477	11.7%	19	8.1%		4.0%
volume (VLBW infants per year)	<30	666	16.3%	37	15.8%	.445	5.6%
	30–56	2171	53.0%	133	56.8%		6.1%
	> = 60	1257	30.7%	64	27.4%		5.1%
type of hospital	university	1495	36.5%	74	31.6%	.088	4.9%
	academic affiliation	2215	54.1%	130	55.6%		5.9%
	other	384	9.4%	30	12.8%		7.8%
neonatal care level	LEVEL I	3948	96.4%	229	97.9%	0.564	5.8%
	LEVEL II	96	2.3%	3	1.3%		3.1%
	Perinatal center	13	0.3%	0	0.0%		0.0%
	Geburtsklinik	37	0.9%	2	0.9%		5.4%
size of department (beds)	< = 17 (= median)	1167	28.5%	66	28.2%	0.917	5.7%
	>17 (= median)	2927	71.5%	168	71.8%		5.7%
size of hospital (beds)	< = 562 (= median)	1578	38.5%	105	44.9%	0.041	6.7%
	>562 (= median)	2516	61.5%	129	55.1%		5.1%

4094 VLBW infants with MICBSI stratified by case fatality risk (CFR). p-values were calculated using Chi-square test. Abbreviations: AGA, appropriate for gestational age; *CoNS*, *coagulase-negative staphylococci*; CVC, central venous catheter (including peripherally inserted central venous catheters and umbilical lines); GW, gestation weeks; LGA, large for gestational age; PVC, peripheral venous catheter; SGA, small for gestational age

In the multivariable analysis, the variable selection was stepwise forward with p = 0.05 for including a parameter in the model and p = 0.10 for excluding a parameter from the model.

The 8 most frequently identified organisms in mono-microbial MCBSI were analyzed: *coagulase negative Stahylococci* (*CoNS*), *S*. *aureus*, *E*. *coli*, *Enterobacter spp*., *Enterococcus spp*., *Klebsiella spp*., *C*. *albicans*, *Serratia spp*.. All other microorganisms were summarized as other mono-microbial MCBSI. While it is common to use,the factor occurring most frequently as reference in a regression model, we decided to use the second most frequent pathogen–S. aureus as reference. This decision was made because the most frequent pathogens, coagulase negative taphylococci are often regarded as contaminants and no real pathogens causing MCBSI [9]. There also was no interaction term included in the model.

In addition, crude and adjusted cumulative survival functions were calculated using Kaplan-Meier method with the time from the last MCBSI to death/discharge/1800g stratified according to the type of microorganism.

The following patient based risk factors were considered in the analysis: birth weight (250g steps: <500g/500g-749g/750g-999g/1000g-1249g/1250g-1499g)), gestational age (GA) (<24 weeks /25-26 weeks /27-28 weeks /29-30 weeks />30weeks), gender (male/female), mode of delivery (planned Caesarean Section/emergency Caesarean Section/vaginal), multiple birth (singleton/multiples), location of delivery (inhouse/admission < = 72h/admission >72h/missing) and time from admission to MCBSI (< = 7/8-14/15-21/> = 22 days). Moreover, small for gestational age (SGA) was defined as birth weight <10th percentile; appropriate for gestational age (AGA) as 10–90% percentile, and large for gestational age (LGA) as >90th percentile based on population-based percentiles, separately for males/females and for singletons/twins [12]. Twin percentiles were used for all multiples because at the time of the study there were no published percentiles for higher-order multiples in the German population. In addition, the following department associated confounder were used in the analysis: care level of the center according to the criteria of the German “Gemeinsamer Bundesausschuss” where Level I is the highest Level and obstetrical hospital is the lowest (I/II/neonatal center, obstetrical hospital), type of hospital (university hospital, academic teaching, others), size of hospital (< = /> 562 beds(median)), size of department (< = />17 beds(median)) and annual volume of VLBW infants (<30/30-59/> = 60).

## Results

A total of 55,465 VLBW infants from 242 neonatal departments were included in the analysis. The overall CFR (until achieving 1800g or transfer from the unit) was 6.4%. 54,204 VLBW infants stayed longer or equal 3 days (CFR 4.8%) with the incidence of MCBSI of 8.1 (infants with > = 1 MCBSI) per 100 VLBW infants and the incidence density of 2.3 MCBSI per 1000 patient days.

**Fig 2:**
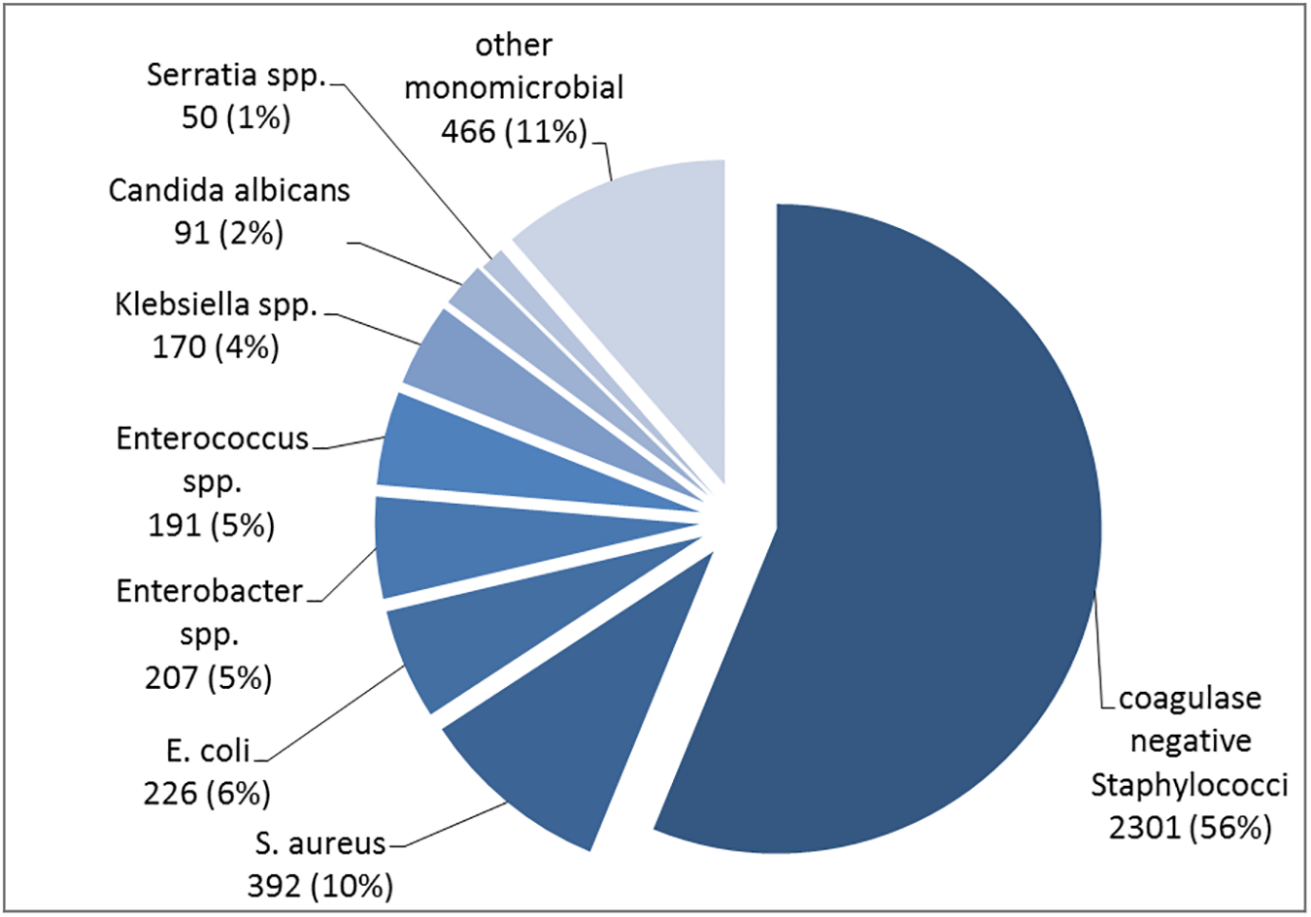
Distribution of monomicrobial bloodstream infections according to the type of pathogen.

**Fig 3:**
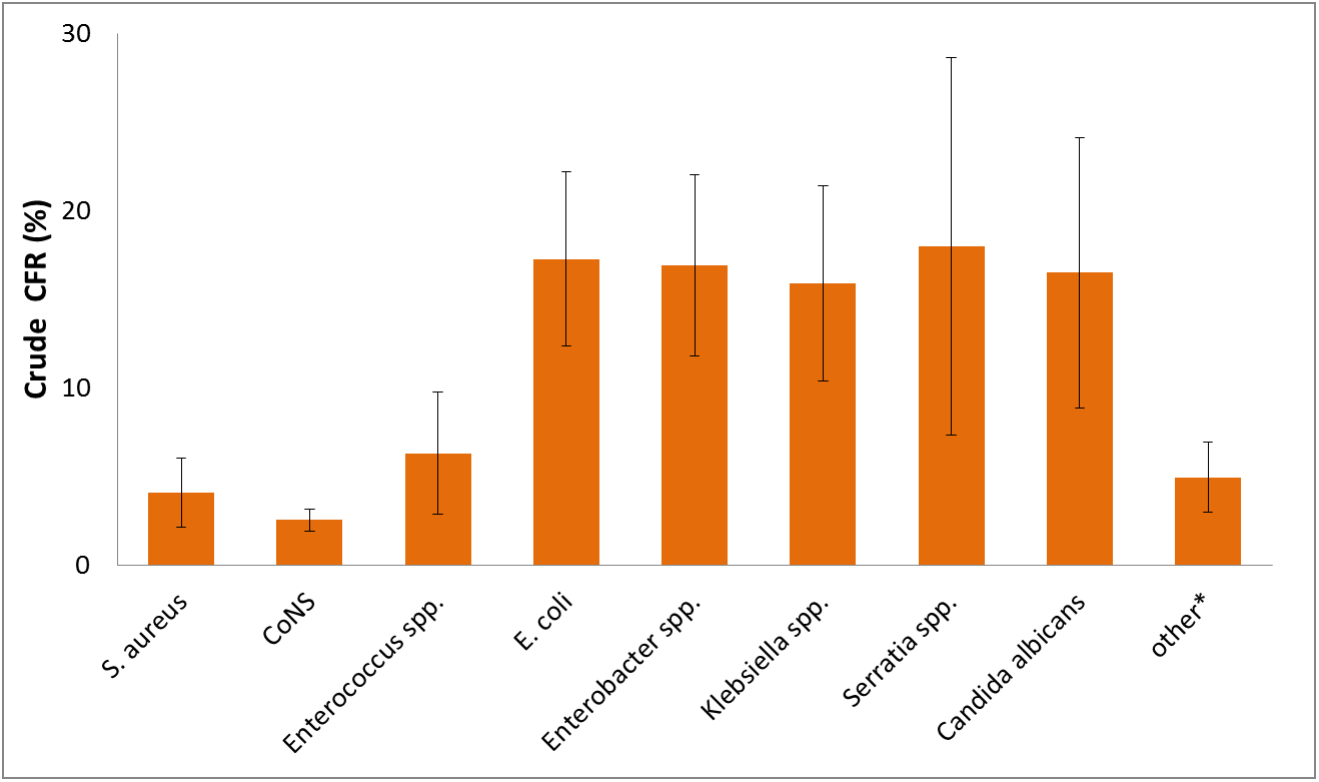
Crude case fatality risk of very low birth weight infants following monomicrobial bloodstream infections according to the type of pathogen. Whiskers represent 95% confidence intervals; *CoNS*, *coagulase negative staphylococci*; other*, other pathogens than listed in the figure.

After considering the exclusion criteria, a total of 4,094 VLBW infants with MCBSI remained (Fig 1). This patient group had an overall CFR of 5.7%. The characteristics of the VLBW infants in the study cohort are described in Table 1. The organisms that were identified most frequently were *CoNS*, followed by *S*. *aureus*, *E*.*coli*, *Enterobacter spp*., *Enterococci*, *Klebsiella spp*., *C*. *albicans* and *Serratia spp*.. All other microorganisms had a percentage of less than 1% each (Fig 2). The crude pathogen specific CFR following MCBSI is shown in Fig 3. The lowest CFR was identified for *CoNS*, followed by *S*. *aureus* and *Enterococci*. The CFR of *E*.*coli*, *Enterobacter spp*., *Klebsiella spp*., *Serratia spp*. and *C*. *albicans* was much higher with minor differences among these microorganisms.

**Fig 4:**
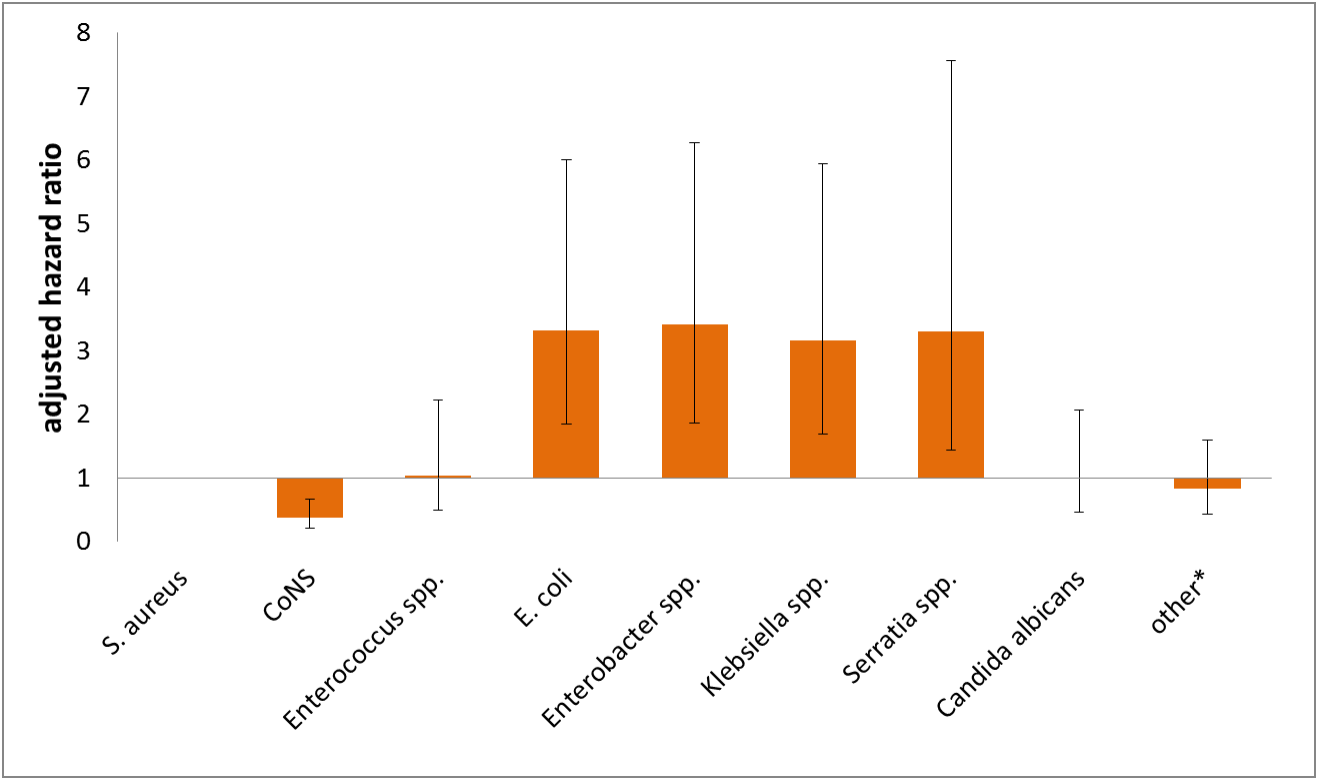
Adjusted hazard ratios for death in very low birth weight infants following monomicrobial bloodstream infections according to the type of pathogen. S.aureus was used as reference value; Whiskers represent 95% confidence intervals; *CoNS*, *coagulase negative staphylococci*; other*, other pathogens than listed in the figure.

**Fig 5:**
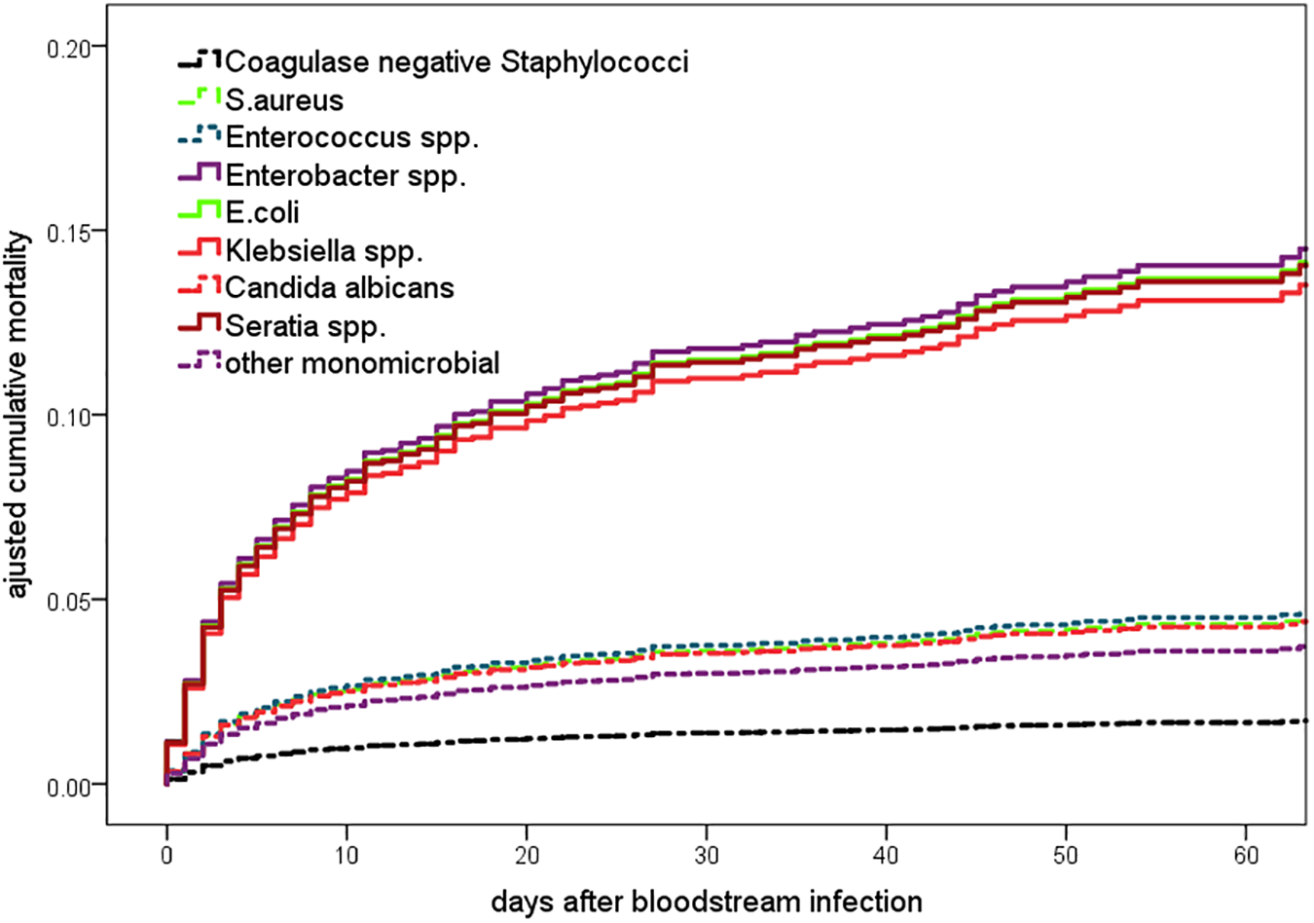
Adjusted cumulative survival function for the time from last monomicrobial blood stream infections to death/discharge/1800g stratified according to the type of pathogen. Adjusted by significant parameters of the multivariable Cox-regression model (see Table 2).

The results of the multivariable Cox proportional hazard regression are shown in Table 2. The multivariate analysis confirmed male gender, birth weight, gestational age, device use, t to infection and birth location as significant risk factors. The Cox proportional hazard regression analysis with adjustment for all available risk factors for death revealed that MCBSI caused by *Klebsiella spp*. (HR 3.17 CI95 1.69–5.95), *Enterobacter spp*. (HR 3.42 CI95 1.86–6.27), *E*. *coli* (HR 3.32 CI95 1.84–6.00) and *Serratia spp*. (HR 3.30 CI95 1.44–7.57) were associated with significantly higher CFR compared to *S*. *aureus*. After adjusting to risk factors, *C*. *albicans* was not a significant risk factor for death. This is also illustrated by Fig 4 which shows the adjusted pathogen specific hazard ratios. The Kaplan Meier survival curve (Fig 5) shows the adjusted survival curves as results of multivariable Cox proportional hazard analysis.

**Table 2:** Results of multivariable Cox proportional hazard regression analysis for death of microbiology confirmed blood stream infections (MCBSI).

Parameter	Category	Total number	Hazard ratio	95%CI	p-value
type of pathogen	*S*. *aureus*	392	1 = reference		<0.001
	*CoNS*	2301	0.38	0.21–0.67	0.001
	*E*. *coli*	226	3.32	1.84–6	<0.001
	*Enterobacter spp*.	207	3.42	1.86–6.27	<0.001
	*Enterococcus spp*.	191	1.04	0.49–2.23	0.918
	*Klebsiella spp*.	170	3.17	1.69–5.95	<0.001
	*C*.*albicans*	91	0.98	0.47–2.07	0.959
	*Serratia spp*.	50	3.3	1.44–7.57	0.005
	other pathogens	466	0.83	0.43–1.59	0.569
gender	male	2216	1.45	1.11–1.91	0.007
birth weight (gramm)	1250–1499	699	1 = reference		<0.001
	1000–1249	782	1.29	0.64–2.59	0.483
	750–999	1136	1.48	0.69–3.17	0.312
	500–749	1208	2.85	1.3–6.28	0.009
	<500	269	4.94	2.11–11.58	<0.001
gestational age (weeks)	>29	771	1 = reference		<0.001
	28–29	932	0.66	0.34–1.27	0.217
	26–27	1022	0.54	0.26–1.12	0.097
	24–25	1119	1.33	0.64–2.77	0.447
	<24	250	1.54	0.69–3.45	0.296
Mode of delivery	CaesareanSection	3297	1 = reference		0.038
	Vaginal	523	1.29	0.92–1.81	0.136
	EmergencyCaesareanSection	274	1.75	1.12–2.73	0.014
Device association	CVC	2029	1 = reference		<0.001
	PVC	1589	0.61	0.43–0.86	0.005
	no device-association	476	0.4	0.24–0.68	0.001
time from admission to BSI (days)	<8	350	1 = reference		<0.001
	8–14	1576	0.47	0.33–0.67	<0.001
	15–21	900	0.25	0.16–0.38	<0.001
	22+	1268	0.21	0.14–0.32	<0.001
Birth Location	Inhouse Birth	3605	1 = reference		0.046
	Immediate Postnatal Transport	130	1.94	1.11–3.4	0.021
	Longterm Postnatal Transport	101	0.71	0.3–1.64	0.418
	n.d.	258	2.3	0.88–6.02	0.089

Abbreviations: AGA, appropriate for gestational age; *CoNS*, *coagulase-negative staphylococci*; CVC, central venous catheter (including peripherally inserted central venous catheters and umbilical lines); GW, gestation weeks; LGA, large for gestational age; PVC, peripheral venous catheter; SGA, small for gestational age

## Discussion

While bloodstream infections in general increase mortality in in VLBW infants [13], this study shows a more than 3 fold increased adjusted CFR of MCBSI caused by gram negative bacteria compared to the CFR of *S*. *aureus* infections. Almost no differences between the specific gram negative microorganisms were detected. With NEO-KISS being a surveillance system that focuses on prevention of infections in neonatology departments, only late-onset infections are recorded, because in most cases the potential for prevention of early-onset infection lies outside the neonatology department. As both pathogen distribution and mortality differs between early-onset and late onset BSI in neonates, this limits our findings to patients with late-onset BSI. While pathogen factors are supposed to be the same for same pathogens in early- and late onset BSI, their interaction with the different physiology of the host in the prepartal phase may lead to different outcomes.

Beside the results concerning the pathogens causing MCBSI, Table 2 shows some additional well-known risk factors for MCBSI and adverse outcomes amongst neonates [9, 14]. Male gender is a well-established risk factor for adverse outcome in preterm neonates[15, 16] as are bad starting conditions like emergency cesarean section[17, 18] and transport to another center in the immediate postnatal period[19–21]. Here the mortality risk in emergency section and postnatal transport may not only come from those unplanned actions themselves but also from the severe patient conditions leading to the respective actions. The fact that PVC use and no use of any intravascular device are protective in our model seem to be a surrogate for relatively better general conditions of the affected children at the time of onset of BSI. Similarly the time from admission to BSI as protective factor is probably due to the age dependent decrease of in hospital mortality in the neonatal population. Finally one might notice that birth weight below 750g significantly increases mortality risk whereas low gestational age is no additional independent risk factor. This may be explained by the approximately linear correlation between gestational age and birth weight in neonates where a deviation from this correlation towards a lower birth weight (small for gestational age) is known to be a risk factor for adverse outcomes including mortality[22].

With follow-up of patients formally ending upon transfer to another department there is a potential risk of losing patients that died in the admitting department for our analysis. While this is a fact and we cannot, by any means, re-identify the patients in the admitting department, we estimate, that this has little impact on the results of our study. NEO-KISS includes all German high level neonatology centers and even almost every German NICU at all, so only very few patients (dying during transport, being transferred to departments in other countries, non-compliance to the surveillance protocol) are not followed up until reaching 1800g or dying.

Our results regarding increased CFR of MCBSI caused by gram-negative bacteria may have implications for the treatment and prevention of BSI in VLBW as probably is worthwhile to focus on gram-negative pathogens from the beginning.

Differences between multi drug resistant organisms (MDRO) and their corresponding drug sensitive counterparts were not investigated in this study, because MDRO infections are still rather rare in the cohort investigated. According to a study from Tsai et al. performed in a NICU in Taiwan, a region with much higher rates of multidrug resistance in gram negative bacteria (18.6%), bacteremia caused by multi drug resistant gram negative bacteria was independently associated with a higher overall case-fatality risk in neonates [8]. Two recent studies, one from the same group, confirmed these findings [6, 7]. This may have an additional impact in the future because of the worldwide increase of antibiotic resistance.

In Taiwan and in another study from Israel, *P*. *aeruginosa* was the pathogen causing the highest CFR of MCBSI [5, 7, 9]. Our study also did not consider *P*. *aeruginosa* as a causative microorganism, because it is a rare pathogen in the German neonatal population which causes less than 1% of MCBSI.

In addition, two of these studies also identified *C*. *albicans* as a significant risk factor for death [8, 9]. While we also observed an increased CFR of *C*. *albicans* MCBSI in our crude analysis, it was equal to that of S. aureus after considering the most important confounding factors. This could be explained by the fact that in our setting the underlying general conditions of patients with *C*. *albicans* infections are worse, but the micro-organism itself is not more virulent. Another factor could be the widespread use of fluconazole prophylaxis [23] in German NICUs which might not prevent infections in all cases but improve the outcome of some infected patients dependent on risk factors.The major strength of our study is the large database with patient parameters allowing for comprehensive adjusting according to the most important risk factors for VLBW. However, our study also has some limitations. We were unable to include appropriateness of initial antibiotic therapy that was administered at the onset of bloodstream infection in the multivariate analysis. As delayed or inappropriate empiric antibiotic therapy is a major driver of mortality this might explain differences when trying to compare our findings to local results. On the other hand, due to the high coverage of German neonatology departments in our surveillance system, our study probably matches the average clinical situation in current German NICUs.

In addition to this limitation, not all conditions of the VLBW at the onset of infection could be considered and information about susceptibility of pathogens to antimicrobial agents was also not included in the analysis as already mentioned.

The significantly higher risk of death following MCBSI due to *E*.*coli*, *Enterobacter spp*., *Klebsiella spp*. and *Serratia spp*. should cause special attention on prevention and treatment of bloodstream infections with these pathogens. While the specific causes of this increase in mortality are still not fully understood, endotoxin release [24] and inappropriate initial therapy are likely to be amongst them. Whether routine microbiological surveillance cultures of patients, that may help to identify colonized patients and to initiate early appropriate treatment and further prevention measures, are an appropriate way to prevent these infections is currently debated in the German neonatological community.

## Conclusion

While we were able to confirm the increased CFR of MCBSI caused by gram-negative pathogens irrespective of the specific species, we could not determine an increased CFR for *Candida albicans* infections compared to *S*. *aureus* after adjusting for common risk factors.

Current strategies for the initial treatment of suspected BSI should focus on the most common gram negative pathogens of the newborn to prevent adverse outcomes. While it seems obvious that the delay in treatment of fungal BSI should influence its CFR this might not be the case in all settings. Further research should be directed towards the outcomes of fungal infections in different settings and under different prophylactic regimens.
